# Efficacy and safety of total glucosides of paeony for rheumatoid arthritis: A protocol for systematic review and meta-analysis

**DOI:** 10.1097/MD.0000000000022224

**Published:** 2020-09-25

**Authors:** Ce Tang, Lianghong Ye, Zhipeng Hu, Wenxiang Wang, Tingting Kuang, Gang Fan, Yi Zhang, XiuHua Liu, Maoyi Yang

**Affiliations:** a Innovative Institute of Chinese Medicine and Pharmacy, Chengdu University of Traditional Chinese Medicine, Chengdu; b Traditional Chinese Medicine hospital. TongLiang. ChongQing, Chongqing; c Hospital of Chengdu University of Traditional Chinese Medicine, Chengdu, Sichuan; d School of Ethnic Medicine.; e School of basic medical sciences, Chengdu University of Traditional Chinese Medicine, Chengdu 611137, China.

**Keywords:** meta-analysis, protocol, Rheumatoid arthritis, systematic review, total glucosides of paeony

## Abstract

**Background:**

Rheumatoid arthritis (RA) is a chronic inflammatory autoimmune disease characterized by erosion of joints and surrounding tissues. RA not only causes the decline of patients’ physical function and quality of life, but also brings huge economic burden to patients’ families and society. Total glucosides of paeony (TGP) is commonly used in treating RA in China. At present, there are many clinical reports about this medicine, but these reports have their own flaws. Therefore, there is an urgent need for systematic review and meta-analysis of the existing clinical evidence.

**Methods and analysis:**

Literature search will be carried out in 6 databases, and the literatures will be screened according to the inclusion and exclusion criteria. The clinical effective rate will be taken as primary outcome. Serum rheumatoid factor, C-reactive protein, erythrocyte sedimentation rate, Western Ontario and McMaster before and after treatment and adverse effects will be secondary outcomes. The heterogeneity of the study will be examined by *χ*^2^ and *I*^2^ test. To identify the source of heterogeneity, subgroup analysis will be carried out. The sensitivity test will be conducted investigate the stability of results. Funnel plot and Egger test will be used to evaluate publication bias. Finally, the quality of evidence will be summarized.

**Results:**

The results will be published in peer-reviewed journals.

**Conclusions:**

This study will systematically evaluate the efficacy of TGP in the treatment of RA. The results of this study can better guide clinical practice.

**OSF registration number:**

DOI 10.17605/OSF.IO/85QVF.

## 1 Introduction

Rheumatoid arthritis (RA) is a chronic inflammatory autoimmune disease characterized by erosion of joints and surrounding tissues.^[1]^ The main pathological changes of RA are chronic synovial inflammation.^[2,3]^ Inflammation invades articular cartilage, subchondral bone, ligaments, tendons, among others, causing destruction of articular cartilage, bone and joint capsule, and various degrees of systemic symptoms.^[4,5]^ The disease is characterized by progressive exacerbation, incurable, and easy recurrence. In China, the disability rates of RA patients in 1 to 5 years, 5 to 10 years, 10 to 15 years, and ≥15 years was 18.6%, 43.5%, 48.1%, and 61.3%, respectively, and the disability rate increases with the duration of the disease.^[6]^ RA not only causes the decline of patients’ physical function and quality of life, but also brings huge economic burden to patients’ families and society. Therefore, how to control RA more effectively and delay its progression has become a hot topic in medical research.

Traditional Chinese medicine (TCM) has a long history and rich experience in the treatment of RA. White peony is a TCM herbal medicine widely used by Chinese medicine doctors in treating RA. It was found that the main active ingredient of paeony is a group of glycosides called total glucosides of white paeony (TGP). At present, TGP has been processed into Chinese patent medicine and widely used in clinical practice. In animal experiments, TGP was found to be able to inhibit inflammation, analgesia, and autoimmune response. These pharmacological effects may be through the inhibition of interleukin-1 (IL-1), tumor necrosis factor-α (TNF-α) and joint synovial cell proliferation, bidirectional regulation of IL-2, and regulation of NF- κB signaling pathway.^[7–9]^ In clinical trials, TGP can downregulate serum rheumatoid factor (RF), C-reactive protein (CRP), erythrocyte sedimentation rate (ESR) levels, reduce inflammatory reaction, and relieve arthritis symptoms. In clinical trials, combination treatments of TGP and other medicines have better efficacy than other drugs alone.^[10]^

However, there are some defects in these clinical trials, such as small sample size, single center research, and low quality. The systematic review of TGP in the treatment of RA did not provide a comprehensive summary and evaluation of the evidence.^[10]^ Therefore, this research will conduct a meta-analysis on the clinical efficacy of TGP in the treatment of RA to provide high-quality evidence for clinical practice.

## 2 Methods

### 2.1 Study registration

We have completed the registration of this study on the Open Science Framework (OSF)(https://osf.io/) and the DOI is 10.17605/OSF.IO/85QVF. The report of this system review protocol is based on the Preferred Reporting Items for Systematic Reviews and Meta-analysis Protocols (PRISMA-P) checklist.^[11]^

### 2.2 Inclusion and exclusion criteria

#### 2.2.1 Study design

Only randomized controlled trials (RCTs) will be included in this study. Non-TCTs, due to their high risk of bias, will not be included in this study.

#### 2.2.2 Participants

Patients with a clear diagnosis of RA will be included in this study. Ideally, the diagnostic criteria should be clearly reported in articles. If the author did not report the diagnostic criteria, we will exclude this research in the main analysis and include it in the sensitivity analysis to assess the impact of the presence or not of the diagnostic criteria on the results. We will not limit other demographic characteristics of participants.

#### 2.2.3 Interventions and comparators

TGP is a kind of Chinese patent medicine, which is made of total glucosides extracted from white peony. At present, the dosage form of TGP on the market is capsule, the standard is 0.3 g, and each capsule contains no less than 104 mg paeoniflorin. We will not limit the usage and dosage of TGP. In addition to TGP, white peony also contains paeonol, volatile oil, and other components. Therefore, the research on the treatment of RA with water extract or ethanol extract of white peony will not be included in this study, whether it is used alone or in combination with other drugs. The control group can be placebo, conventional treatment, and care or no treatment. Studies that compare TGP with other traditional Chinese herbal medicine will not be included.

#### 2.2.4 Outcomes

Primary outcome: clinical effective rate.

Secondary outcomes: serum rheumatoid factor (RF), CRP, ESR, Western Ontario and McMaster before and after treatment; adverse effects.

#### 2.2.5 Other criteria

Conference articles and abstract articles will not be included in this study because there are no data to be analyzed. For these articles, we will classify them as awaiting classification, and contact the authors for detailed research data.

### 2.3 Study search

The following databases will be searched from its inception to August 2020: PubMed, Embase, Cochrane Library Central Register of Controlled Trials, and 4 Chinese databases: China National Knowledge Infrastructure database, Wanfang Data Knowledge Service Platform, and the VIP information resource integration service platform. There will be no language restriction. We will also search the Chinese clinical trial registry (ChiCTR) and ClinicalTrials.gov to find ongoing research. In addition, we will search the references of the included articles to find more citations. The search strategy of PubMed can be viewed in Table 1.

**Table 1:**
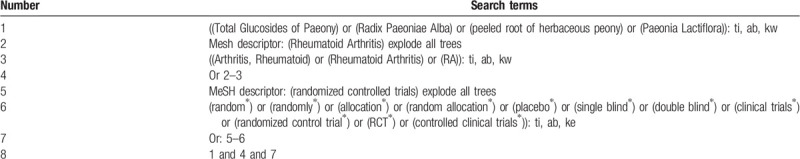
Search strategy in PubMed.

### 2.4 Study selection

**Figure 1:**
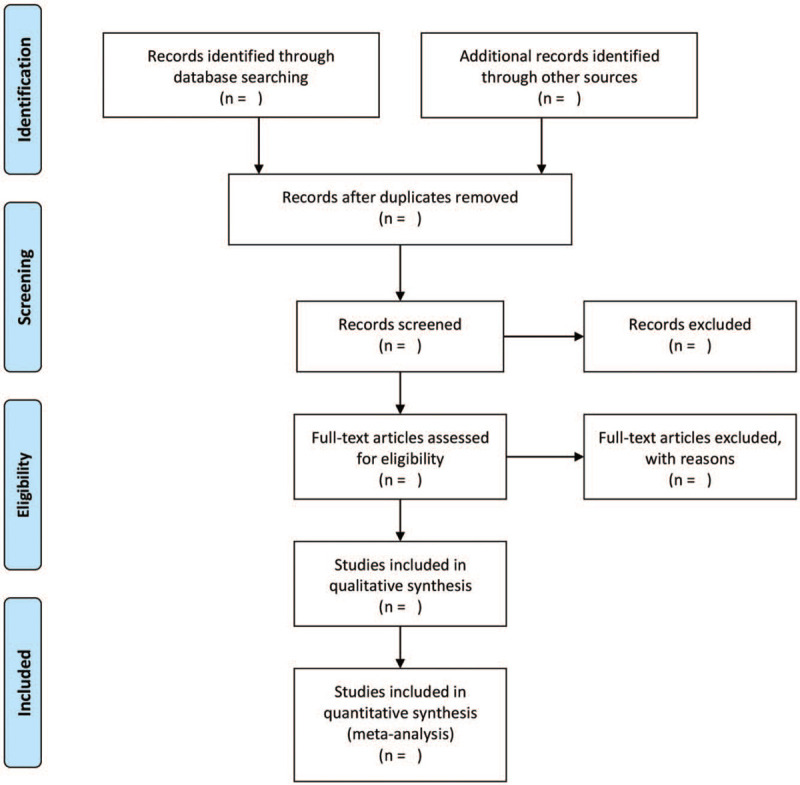
Flow chart of study selection.

Two authors will screen the literature by reading the title and abstract independently. We will download the full text of all possible eligible literatures. Any differences in the research process will be resolved through consultation with a third researcher. If it is still unable to decide whether to include a study or not after negotiation, the study will be classified as awaiting classification. We will draw a flow chart and provide a list of excluded literature and reasons for exclusion (Fig. 1).

### 2.5 Data extraction

We will extract the data from the literature by using a pre-specified form. The following data will be extracted: first author name, publication time, country, type of study, number of trial group, number of control group, main outcome, secondary outcomes, and adverse events. We will contact the author for more information if necessary. Data extraction will be carried out independently by 2 authors.

### 2.6 Risk of bias assessment

The risk of bias of included studies will be assessed by using the latest version 2 of the Cochrane risk-of-bias tool for randomized trials (RoB2).^[12,13]^ We will assess the risk of bias of primary outcome of the research. The nature of the effect of interest was “intention-to-treat” effect. The bias risk assessment will be conducted by MY and ZH independently, and inconsistencies will be solved by consensus. This tool has 5 domains: bias arising from the randomization process, bias due to deviations from intended interventions, bias due to missing outcome data, bias in measurement of the outcome, and bias in selection of the reported result. Each domain has 3 judgments: low risk of bias, some concerns, and high risk of bias. The results will be represented as “traffic light” plots and weighted bar plots.

### 2.7 Data analysis

R studio Version 1.2.1335 will be used for data analysis. We will pool data that do not have significant clinical heterogeneity. Binary variables will be represented by risk ratio (RR) and 95% confidence interval (CI) and continuous variables will be represented by mean difference (MD) and 95% CI.^[14]^ The statistical heterogeneity will be identified by using *χ*^2^ test with significance level of α = .1. At the same time, we will use *I*^*2*^ statistics to quantify the size of heterogeneity. *I*^*2*^ ≥50% will be considered as significant heterogeneity.^[15]^ If quantitative synthesis is not appropriate, the results will be presented as tables.

### 2.8 Subgroup analysis

We will conduct subgroup analysis based on pre-set subgroup hypotheses to explore the heterogeneity between studies. Subgroup hypotheses include the following: gender (male/female), control group (no treatment or placebo/conventional treatment), treatment period (depending on data).

### 2.9 Sensitivity analysis

We will conduct sensitivity analysis to explore whether the results are robust. We will carry out sensitivity analysis by using different effect measures (RR or OR) and statistical models (fixed effect model or random effects model). In addition, we also included studies without clear diagnostic criteria into the analysis to explore the impact of the presence or not of diagnostic criteria on the research results.

### 2.10 Publication bias

If >10 studies are included, we will conduct publication bias assessment. We will assess publication bias by funnel chart and quantify publication bias by Egger test. Funnel plot is asymmetric or *P* < .05 in Egger test indicates the existence of publication bias.

### 2.11 Certainty of evidence

Finally, we will evaluate the certainty of the evidence by using Grading of Recommendations Assessment, Development and Evaluate system (GRADE). The quality of the evidence will be rated as high, medium, low, or very low. Finally, the main results of this result will be presented as a summary of table.

### 2.12 Patient and public involvement

No patients and public will be involved in the study.

### 2.13 Ethics and dissemination

Ethical approval is not needed. Our findings will also be published in peer-reviewed journals.

## 3 Discussion

RA is a chronic and recurrent rheumatic immune disease. In China, TCM is often used as an adjuvant treatment for RA and TGP is one of the representative drugs. The current systematic review of the drug in the treatment of RA has flaws. Therefore, this study will comprehensively summarize and evaluate the evidence for the treatment of RA with GLP. To ensure the quality of the methodology, this study will use the latest version of the risk of bias assessment tool. In addition, we will evaluate the certainty of the evidence and present the results as summary of table, which will be more conducive to the clinical application of the results of this study.

### 3.1 Amendments

In the process of research, if there is any need to modify our plan, we will update our plan in time.

## Author contributions

**Conceptualization:** Ce Tang , Lianghong Ye.

**Data curation:** Zhipeng Hu, Maoyi Yang.

**Formal analysis:** Wenxiang Wang, Tingting Kuang.

**Investigation:** Gang Fan, Yi Zhang.

**Methodology:** Maoyi Yang, Zhipeng Hu.

**Project administration:** XiuHua Liu.

**Software:** Ce Tang.

**Visualization:** Lianghong Ye, Zhipeng Hu.

**Writing – original draft:** Ce Tang.

**Writing – review & editing:** Maoyi Yang.
